# Bilateral synchronous tonsillar metastasis from a mixed form of neuroendocrine tumor in the lung: Case report and literature review

**DOI:** 10.1111/1759-7714.13714

**Published:** 2020-10-26

**Authors:** Antoine Noël, Alexandra Rodriguez, Clément Lelong, Jean‐Christophe Noël

**Affiliations:** ^1^ Department of Otorhinolaryngology ‐ Head and Neck Surgery Erasme University Hospital, Université Libre de Bruxelles Brussels Belgium; ^2^ Department of Otorhinolaryngology ‐ Head and Neck Surgery C.H.U Saint‐Pierre, Université Libre de Bruxelles Brussels Belgium; ^3^ Department of Pathology Erasme University Hospital, Université Libre de Bruxelles Brussels Belgium

**Keywords:** Cell transdifferentiation, lung cancer, neuroendocrine carcinoma, palatine tonsils

## Abstract

Here, we report the case of a 54‐year‐old man with a history of squamous cell carcinoma (SCC) of the lung, who developed bilateral neuroendocrine carcinoma (NEC) of the palatine tonsils. Faced with this atypical situation, another biopsy of the lung lesion was performed, revealing NEC histology patterns. This article describes the first case of metastasis to the bilateral palatine tonsils from the NEC component of a mixed NEC/SCC of the lung, highlighting the importance of reconsidering the diagnosis of the primary tumor histology, particularly in lung cancer with the possible presence of mixed tumor after phenotype transdifferentiation of the primary tumor.

**Key points:**

**Significant findings of the study:**

Mixed lung carcinoma can be revealed after the presence of neuroendocrine carcinoma metastasis.

**What this study adds:**

Bilateral neuroendocrine carcinoma of the palatine tonsils should be considered as metastases, particularly in the presence of lung cancer with a poor response to treatment.

## Graphical abstract

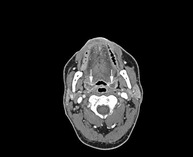

Mixed lung carcinoma can be revealed after the presence of neuroendocrine carcinoma metastasis.Bilateral neuroendocrine carcinoma of the palatine tonsils should be considered as metastases, particularly in the presence of lung cancer with a poor response to treatment.

## Introduction

Metastatic disease of the tonsils is very uncommon and accounts for approximately 0.8% of all tonsillar malignancies. Only 22 cases of lung cancer with unilateral palatine tonsillar metastasis have been previously reported in the literature.^1^

Here, we report the first case of bilateral palatine tonsillar metastasis from the neuroendocrine carcinoma (NEC) component of a mixed NEC/SCC of the lung in a 54‐year‐old man.

## Case report

A 54‐year‐old man with a history of long‐term smoking, was diagnosed with a cT3N3M1a (stage IVA) squamous cell carcinoma (SCC) of the lung with metastatic involvement of the pulmonary pleura. He was treated with chemotherapy (cisplatin‐gemcitabine). In view of the poor response to treatment and regional disease progression with metastasis to the pelvis, immunotherapy was initiated.

Two years later, progression of disease resulted in new brain metastases that were treated by gamma knife stereotactic radiosurgery.

**Figure 1:**
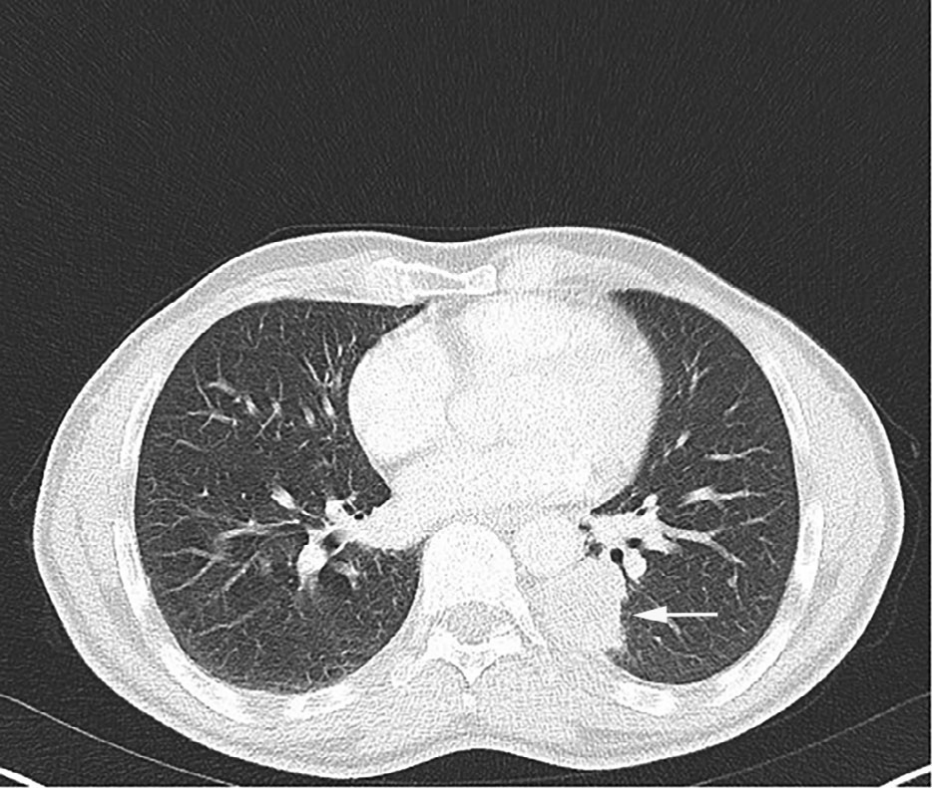
Chest computed tomography (CT) revealed progression of the tumor in the left lung (white arrow).

**Figure 2:**
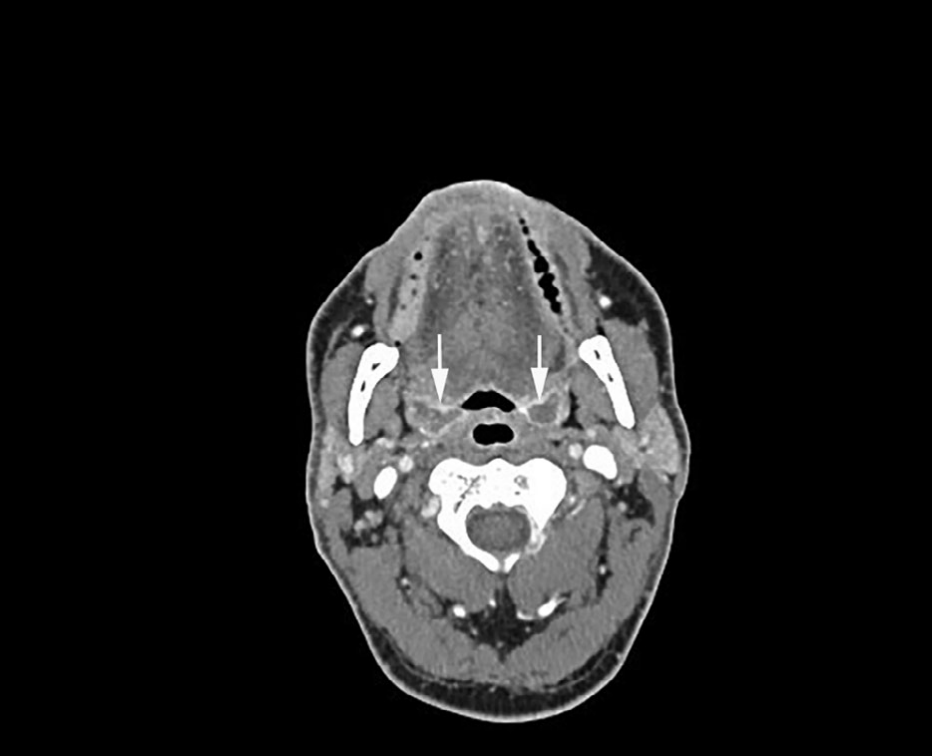
Head and neck computed tomography (CT) revealed involvement of the bilateral palatine tonsils (white arrows).

A few months later, the patient was referred to the head and neck surgery department for pharyngeal discomfort which had been present for several weeks. Oropharyngeal and laryngeal examination revealed bilateral moderate enlargement of the palatine tonsils with areas of ulceration. Computed tomography (CT) scan and positron emission tomography (PET) scan showed the progression of the lung carcinoma (Fig 1) with suspected carcinomatous involvement of the left cervical lymph nodes and bilateral palatine tonsils (Fig 2). Histopathological examination of a biopsy of the left tonsil revealed a lesion with features highly suggestive of carcinoma.

**Figure 3:**
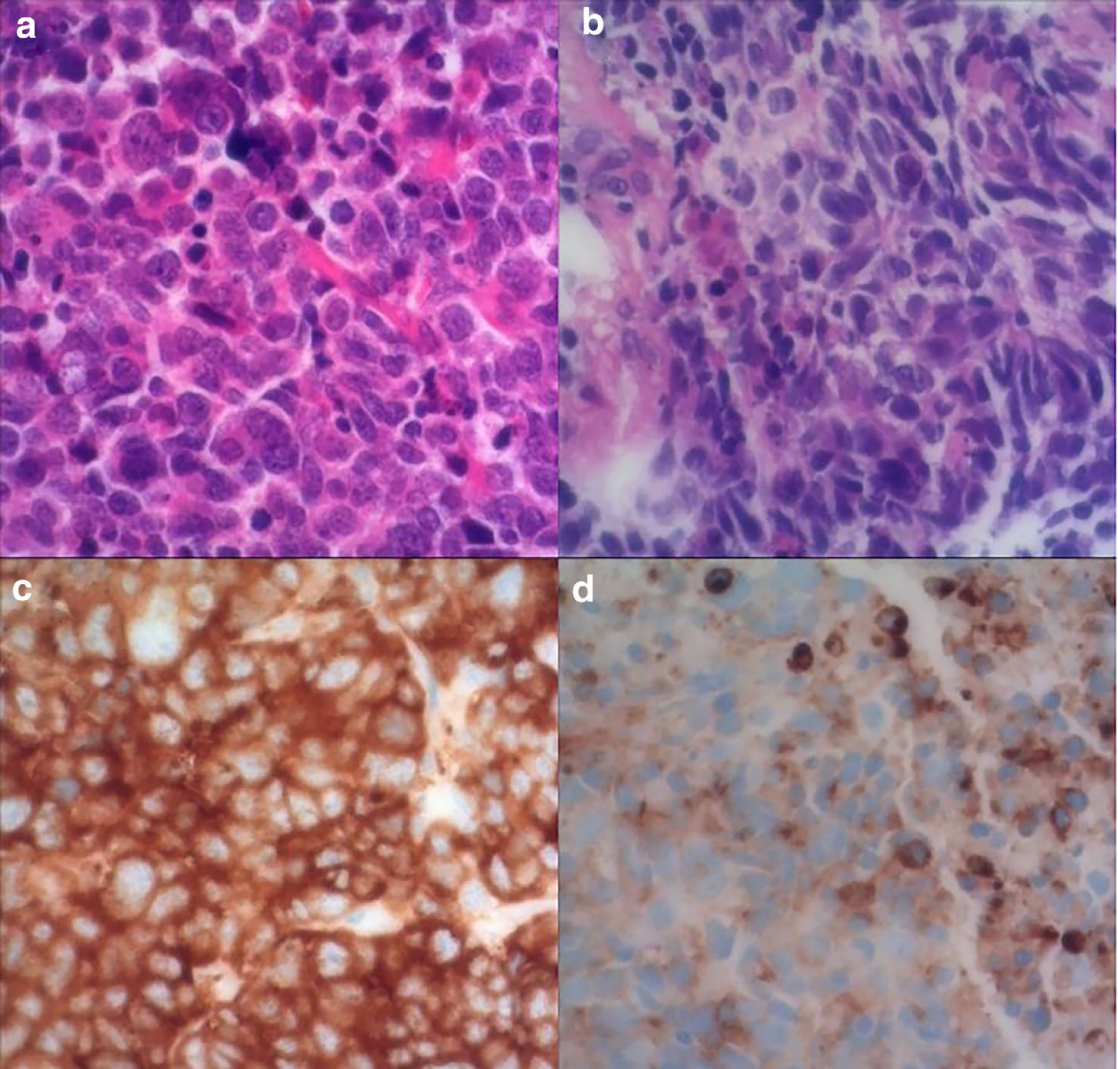
Photomicrography of tumor sections. (**a**) Hematoxylin and eosin staining of the surgically excised tonsil lesion (magnification × 400). (**b**) Hematoxylin and eosin staining of a biopsy specimen from the left lung (magnification × 400). (**c**) Immunohistochemistry of the tumor cells revealed synaptophysin positivity (magnification × 400). (**d**) Immunohistochemistry also revealed tumor cells that were positive for synaptophysin (magnification × 400).

Bilateral transoral partial oropharyngectomy with neck dissections was performed, revealing two distinct tonsillar lesions with NEC histology and metastatic involvement of two left cervical lymph nodes. In light of these findings, a second transbronchial biopsy at the primary lung tumor was performed, which revealed a NEC with identical histopathological features to those of the oropharyngeal carcinoma, confirming that the palatine tonsil lesions were metastases from NEC components (Fig 3) of a mixed NEC/SCC of the lung.

Following discussion by the multidisciplinary oncology group, the patient received six courses of cisplatin and etoposide chemotherapy. The six‐month follow‐up showed progression of the lung carcinoma with liver metastasis. The patient died several months later.

## Discussion

NEC represents only 0.5% of all malignant tumors and the second most common site of NEC is in the lungs.^2^ The oropharynx is an extremely rare primary site of NEC, representing only 0.03% of all head and neck cancers.^3^

The presence of a new bilateral tonsillar NEC with atypical histological findings therefore requires careful review before considering it to be a new synchronous‐metachronous primary lesion for several reasons. First, in the literature, the presence of bilateral synchronous tonsillar carcinoma only concerns SCC and has been reported in 2.3% of cases^4^ and no case of primary bilateral neuroendocrine carcinoma has ever been described. Second, 60% to 70% of cases of poorly differentiated NEC are associated with distant metastases at the time of diagnosis^5^ and NEC is revealed by metastases in 12%–22% of cases.^2^ Therefore, in the concomitant presence of lung carcinoma, bilateral tonsillar lesions are more likely to correspond to metastases, particularly in a patient with a poor response to treatment.

The main confounding factor in this clinical case was the initial diagnosis of SCC of the lung rather than mixed NEC/SCC of the lung. Due to the rarity of metastases in the palatine tonsils, which account for 0.8% of all malignancies of the tonsils,^1^ this atypical situation raised the suspicion of two distinct primary carcinomas: a primary SCC of the lung and a metachronous NEC of the palatine tonsils. The presence of a mixed NE/non‐NE carcinoma of the lung is rare with an estimated incidence of 2.5 to 5 per 100 000 people per year.^6^ The presence of a mixed lung carcinoma can be explained by the marked plasticity of lung carcinoma to change phenotype.^7^ A retrospective study on 22 mixed NE/non‐NE carcinomas of the lung revealed three cases of mixed NEC/SCC of the lung.^6^ No case of histological transdifferentiation from SCC to NEC has been reported in the literature. As illustrated in the case reported by Watanabe *et al*. the primary carcinoma in our case may actually have been a NEC that transdifferentiated to squamous cell carcinoma^7^ and, at the time of the first lung biopsy, the SCC component was probably predominant, while the NEC component was missed.

Due to the rarity of this disease, optimal management has not been clearly defined. The clinically aggressive nature of neuroendocrine tumors must be rapidly identified, because treatment and prognosis are based on the grade of the NEC and the extent of disease.^5^ The first‐line treatment for metastatic NEC of the lung consists of platinum‐based chemotherapy (cisplatin/etoposide), but the prognosis remains poor with a mean life expectancy of less than seven months in patients with metastases.^8^

In conclusion, the presence of bilateral NEC of the palatine tonsils should be considered to be metastases from another carcinoma until proven otherwise, particularly in a patient with a poor response to treatment. This is particularly true in the presence of lung cancer with the possible presence of mixed tumor after phenotype transdifferentiation of the primary tumor.

## Disclosure

No authors report any conflict of interest or financial support for this work.
